# Analysis of clinical pharmacist interventions in the neurology unit of a Brazilian tertiary teaching hospital

**DOI:** 10.1371/journal.pone.0210779

**Published:** 2019-01-18

**Authors:** João Paulo Vilela Rodrigues, Fabiana Angelo Marques, Ana Maria Rosa Freato Gonçalves, Marília Silveira de Almeida Campos, Tiago Marques dos Reis, Manuela Roque Siani Morelo, Andrea Fontoura, Beatriz Maria Pereira Girolineto, Helen Palmira Miranda de Camargo Souza, Maurílio de Souza Cazarim, Lauro César da Silva Maduro, Leonardo Régis Leira Pereira

**Affiliations:** 1 Pharmaceutical Services and Clinical Pharmacy Research Center, School of Pharmaceutical Sciences of Ribeirão Preto, University of São Paulo, Ribeirão Preto, Brazil; 2 Clinical Analysis Department, Federal University of Alfenas, Alfenas, Brazil; 3 Health Sciences Center, Federal University of Piauí, Piauí, Brazil; Drake University College of Pharmacy and Heath Sciences, UNITED STATES

## Abstract

It is estimated that around five to 10.0% of hospital admissions occur due to clinical conditions resulting from pharmacotherapy. Clinical pharmacist's activity can enhance drug therapy's effectiveness and safety through pharmacotherapy interventions (PIs), thus minimizing drug-related problems (DRPs) and optimizing the allocation of financial resources associated with health care. This study aimed to estimate the DRPs prevalence, evaluate PI which were performed by clinical pharmacists in the Neurology Unit of a Brazilian tertiary teaching hospital and to identify factors associated with the occurrence of PI-related DRP. A single-arm trial included adults admitted in the referred Unit from 2012 July to 2015 June. Patients were evaluated during their hospitalization period and PIs were performed based on trigger DRPs that were detected in medication reconciliation (admission or discharge) or during inpatient follow-up. Student's t-test, Chi-square test, Pearson and Multiple logistic regression models to analise the association among age, number of drugs, hospitalization period, and number of diagnoses with occurrence of DRPs. Analyses level of significance was 5%. In total 409 inpatients were followed up [51.1% male, mean age of 49.1 (SD 16.5)]. Patients received, on average, 11.9 (SD 5.8) drugs, ranging from two to 38 drugs per patient, and 54.3% of the sample presented at least one DRP whose most frequent description was "untreated condition". From all 516 performed PIs that resulted from DRPs, 82.8% were accepted and the majority referred to "drug introduction" (27.5%). Multiple logistic regression showed that age, length of hospital stay, number of drugs used, diagnosis of epilepsy, multiple sclerosis and myasthenia gravis would be clinical variables associated with DRP (*p* < 0,05). Monitoring the use of drugs allowed the clinical pharmacist to detect DRPs and to suggest interventions that promote rational pharmacotherapy.

## Introduction

Inappropriate use of medication constitutes a major public health issue that negatively affects treatment response and increases costs regarding the management of drug-related problems (DRPs). DRP refers to drug treatment events which may interfere its results, being a frequent cause of morbidities, hospitalizations and mortality. They can be associated to aspects, such as indication need, treatment effectiveness, safety, which includes detection and prevention of adverse drug reactions, and drug therapy adherence [1,2].

Moreover, around 5.0 to 10.0% of hospital admissions are estimated to occur due to DRPs, from which up to 60.0% are preventable [3,4]. The clinical pharmacist is recognized for acting together with other health team professionals and patients, and for performing pharmacotherapy interventions (PIs) that enhance drug therapy's effectiveness and safety [5,6]. Thus, by reducing the incidence of DRPs, clinical pharmacy services (CPSs) are able to optimize the use of financial resources associated with the provision of inpatient health care [7–10].

Considering the hospital setting, clinical pharmacist's performance should occur during the whole hospitalization period, from admission to discharge. At these specific two moments, it is recommended to perform the medication reconciliation, a practice whose purpose is to review and evaluate if current medical prescriptions are coherent with the previous prescriptions, and also with medical history. Hence, it is possible to detect divergencies that may impact on clinical evolution of patients and impair their health [11].

Among the potential patients for CPSs, it is noteworthy that individuals who are diagnosed with neurological diseases are more susceptible to the occurrence of DRPs, once medications indicated to manage most common conditions have complex dosage regimens, potential for interaction with other drugs and/or are associated with the occurrence of important adverse reactions [12,13]. There is evidence that the inclusion of clinical pharmacist into health care teams which provide care in the context of diseases such as epilepsy and Parkinson's disease improves clinical outcomes and quality of life of assisted patients in response to PIs performed from potential or actual DRPs [14–16].

From this backgroung, this study aimed to estimate the DRPs prevalence, evaluate PIs which were performed by clinical pharmacists in the Neurology Unit of a quaternary teaching hospital, and to identify factors associated with the occurrence of PI-related DRP.

## Patients and methods

We carried out a single-arm trial at the adult Neurology Unit of the General Hospital of Medical School of Ribeirão Preto, University of São Paulo, Brazil (HCFMRP-USP). HCFMRP-USP is a tertiary teaching hospital focused on teaching, researching, and assisting Brazilian Public Health System patients. Inpatients medical prescriptions, as well as their clinical and laboratorial information were accessed through the hospital's eletronic information system. Regarding Neurology Unit, there are 26 beds for the hospitalization of adults with previously diagnosed neurological disease or for diagnostic investigation.

The inclusion criteria embraced individuals of both sexes, aged 18 years or more, who were admitted in the adult Neurology Unit of HCFMRP-USP between 2012 July 1st and 2015 June 30th. We did not include patients who were hospitalized exclusively to undergo polysomnography examination, as well as individuals whose hospitalization lasted less than 24 hours and those who did not agree with the Informed Consent Form. All patients included signed the Informed Consent. Sample size calculation was performed through a prevalence formula [17], and was based on average prevalence of health team adherence to pharmacists PI, regarding five previous studies carried out in a similar context to the present study [18–22]. We considered a level of significance (α) of 5% for an infinite population. Therefore, the minimum sample size required would be 134 individuals.

**Fig 1:**
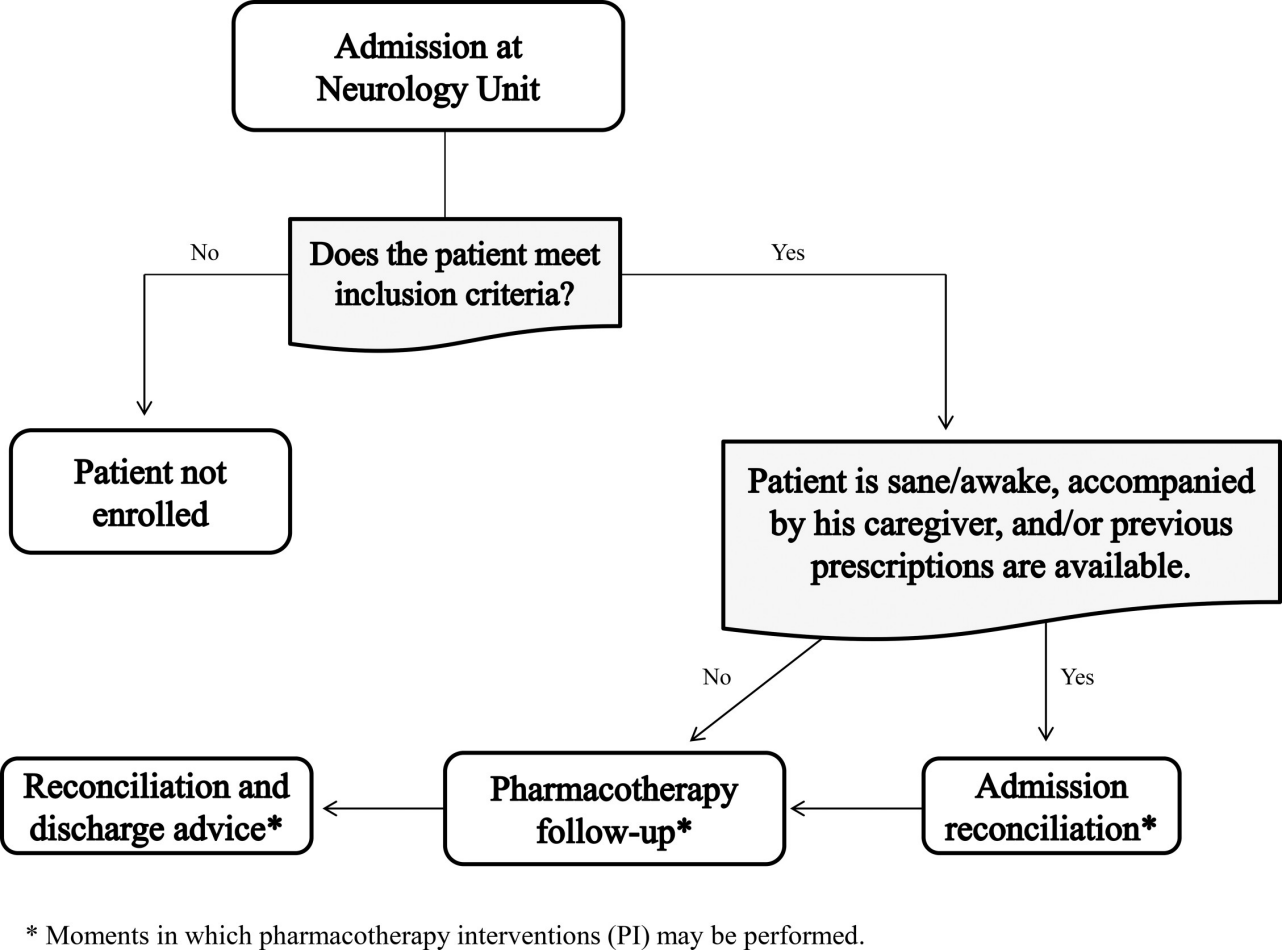
Summarizing algorithm for the care process offered by the pharmacist during the hospitalization of patients in the Neurology Unit of HCFMRP-USP. * Moments in which pharmacotherapy interventions (PIs) may be performed.

The CPS that was performed from Monday to Friday by one researcher pharmacist takes place in three steps: reconciliation on admission, follow-up based on daily pharmacotherapy review and e hospital discharge reconciliation. Patient follow-up and information collection through structured CPS forms began at hospital admission (Fig 1). The main objective of the professional's activity at this moment was to record patient's clinical history mainly related to the use of drugs at home. If it was detected any inconsistency between collected information and hospital medical prescription, the pharmacist did intervene through a PI to the physician in order to solve the discrepancy or DRP. Admission medication reconciliation occurred through direct contact with the patient and/or caregiver and also by means of analysing other prescriptions brought by the patients, regarding their home treatment routine.

From the admission medication reconciliation until hospital discharge, the collection and examination of clinical data from each patient was performed on a daily basis, as well as the pharmacotherapy follow-up. Information about the clinical condition was recorded as suggested by the SOAP method (Subjective-Objective-Assessment-Plan) [23–25]. Subjective data refer to symptoms based on patient's main complaints. The pharmacist then recorded objective data, such as laboratory tests results, blood pressure, heart rate and body temperature.

Pharmacotherapy follow-up activities focused on issues related to patient's need, as well as to treatment effectiveness and safety. Therefore, a careful analysis of medical prescription was undertaken with respect to indication, dose, effectiveness, adverse drug reactions, drug-drug and drug-food interactions, interactions between drug and enteral nutrition, among others.

After subjective and objective collection data and daily prescription analysis, the pharmacist proceeded to the case evaluation and to the establishment of an intervention plan. These steps were registered into the clinical evolution form, according to the SOAP method.

Medication reconciliation at discharge was performed by reviewing both patient's medical history and medical prescriptions evolution, and then counseling was provided to the patient and/or his caregiver. If any inconsistency was detected between the previous medical prescriptions and the discharge prescription, the pharmacist then formulated a PI addressed to the physician in order to solve the DRP. During patient and/or caregiver advice, following medication reconciliation, the pharmacist delivered information on access, storage, and use of drugs. This occurred verbally, and also written through tools such as tables and pictograms, in order to assure interlocutors understanding. Thus, at hospital discharge, the pharmacist could perform two types of interventions: intervention to the physician as a result of a DRP detected and intervention with patient/caregiver related to education about the correct use of drugs at home. Intervention related to education was executed even if there was not a DRP. Data from these practices were recorded in specific forms for discharge medication reconciliation.

All PIs carried out during pharmaceutical follow-up, admission reconciliation and discharge medication reconciliation were conducted verbally and registered into the electronic patient record. Categorization of performed PI that result from a DRP was made according to the trigger DRP classification (Table 1), which was adapted from Cipolle, Strand and Morley (2004) [26], to match the reality of this study's CPS. DRP that refers to non adherence to pharmacotherapy was not considered because in the hospital where the study was performed the nursing team administers or supervises the administration of drugs. Therefore, adherence to the pharmacological treatment by the patient does not constitute a possible DRP during hospitalization.

**Table 1:** Types of DRPs and PIs performed by clinical pharmacist from the CPS in the Neurology Unit of HCFMRP-USP.

DRP classification	DRP description or cause	Feasible PI based on detected DRP
Need for indication	Untreated condition	Drug introduction
	Unnecessary treatment	Drug withdrawal (unnecessary or duplicated)
Effectiveness	Ineffective or potentially ineffective drug	Dose increase
		Drug substitution
		Introduction of a new drug
		Administration schedule change
		Administration route change (same drug)
	Drug interaction	Withdrawal of a drug due to decreased effect of another.
		Increase in dose of drug whose effect is reduced
		Dose decrease of drug that reduces the effect of the other
		Administration schedule change
		Drug substitution
		Introduction of a new drug
	Drug-food interaction	Drug or food administration schedule change
	Subtherapheutic dosage^1^	Dose increase
		Extension of treatment period
		Increase of i.v.^5^ drug infusion rate
	Drug-enteral feeding tube interaction	Drug substitution
		Pharmaceutical formulation change (same drug and administration route)
		Administration route change (same drug)
	Physical-chemical incompatibility^2^	Administration schedule change
		Dose/concentration decrease
		Diluent substitution
		Administration route change
Safety	ADR^3^/Potential ADR/Contraindication	Drug substitution
		Drug withdrawal
		Dose decrease
		Administration schedule change
		Administration route change
		Drug introduction for ADR management / prevention
	Overdosage^4^	Daily dosage decrease
		Reduction of treatment period
		Decrease of i.v.^5^ drug infusion rate
	Drug interaction	Withdrawal of the drug which is causing ADR
		Dose decrease of the drug which is causing ADR
		Withdrawal of drug that increases serum level of another
		Dose decrease of the drug that increases serum level of another
		Dose decrease of one of the drugs due to synergistic effect
		Dose decrease of both drugs due to synergistic effect
		Drug substitution
	Physical-chemical incompatibility^2^	Administration schedule change
		Dose / concentration decrease
		Diluent substitution
		Administration route change
Other	Laboratory monitoring not performed^6^	Laboratory test order
	Cost/Access^7^	Drug substitution
		Administration route change (same drug)
		Change of marketed formulation (same drug and pharmaceutical formulation)
	Complex pharmacotherapy regimen^8^	Administration schedule change
		Change of marketed formulation (same drug and pharmaceutical formulation)
	Other	Education / discharge advice
		Other

DRP: drug-related problem; PI: pharmacotherapy interventions; CPS: clinical pharmaceutical service.

^1^Dose is lower than the minimum recommended by the literature for the correspondent indication

^2^Drugs or drug and diluent which are prescribed for i.v. administration but their concomitant use through i.v. route is contraindicated. Precipitate or insoluble complexes formation may occur, resulting for example in ineffectiveness and / or adverse events to the patient

^3^ADR: adverse drug reaction

^4^Dose is higher than the upper established limit for the respective indication or condition. It occurs, for instance, in cases where renal damage is present but the recommended dose adjustment has not been performed

^5^i.v.: intravenous route

^6^Pharmacist suggests to the physician the request of laboratory tests to monitor pharmacotherapy effectiveness/safety

^7^Pharmacist suggests the change of either the drug, the administration route, or the pharmaceutical formulation, aiming to minimize the costs after hospital discharge for both the health system and patient

^8^This results in an intervention in order to provide the patient a more rational and convenient treatment scheme. An example would be the suggestion to adjust the administration schedule so that some drugs can be taken at the same time—as long as there are no drug interactions that contraindicate it.

It is importante to highlight that one single DRP may result in different PIs that will be performed at the same time. A safety DRP, for example, may result in both drug withdrawal and introduction of a new drug to manage the respective adverse reactions signs and symptoms.

The pharmacist recorded each PI and the description of its trigger DRP in specific form, and also at what moment the DRP was detected–by admission, during the hospitalization or by discharge. PI described as “Education/Discharge advice” refers to the aforementioned counseling which was provided to patient and/or caregiver after discharge medication reconciliation.

In order to identify factors associated with the occurrence of DRPs, the following variables were considered: sex, age, hospitalization period, etiological diagnosis related to neurological disorder, number of diagnosed conditions, and number of prescribed drugs. Additionally, drugs related to PI were classified according to the following systems: Anatomical Therapeutic Chemical (ATC), and Defined Daily Dose (DDD) [27]. It is emphasized that each hospitalization was considered as an independent patient, that is, a single patient who underwent two hospitalizations during the study period was as two different patients. Quantitative variables were expressed as the mean and respective standard deviation (SD), while qualitative variables as absolute and relative frequencies. Regarding analytical statistics, patients were divided into the categories "with DRP" and "without DRP".

Then, unpaired Student's t-test was performed to compare the categories' means with respect to hospitalization period and age, Chi-square test (χ2) was used to identify the association between patient's sex and the occurrence of DRP, and finally, we calculated Poisson model to compare number of diagnoses and number of prescribed drugs during hospitalization. Multiple logistic regression model was employed to verify the adjusted association among: most common etiological diagnoses that led to hospitalization at neurology ward, age, number of prescribed drugs, length of hospital stay (days), and number of diagnoses with occurrence of DRPs through the adjusted Odds Ratio. Analyses level of significance (α) was set at 5% and were delevoped through Statistical Package for Social Sciences Program (SPSS Inc., version 17.1.0).

This study was approved by the Research Ethics Committee of HCFMRP-USP, approval number 1888333.

## Results

A total of 409 adults admitted to the Neurology Unit of HCFMRP-USP were followed up, with a mean age of 49.1 years (SD 16.5). Regarding gender, men composed 51.1% of the sample. Among all patients, 222 (54.3%) presented at least one DRP during the hospitalization period; adding up to 516 DRPs, a mean of 2.3 (SD 2.1) per patient, and a minimum and maximum value of one and 14, respectively.

**Fig 2:**
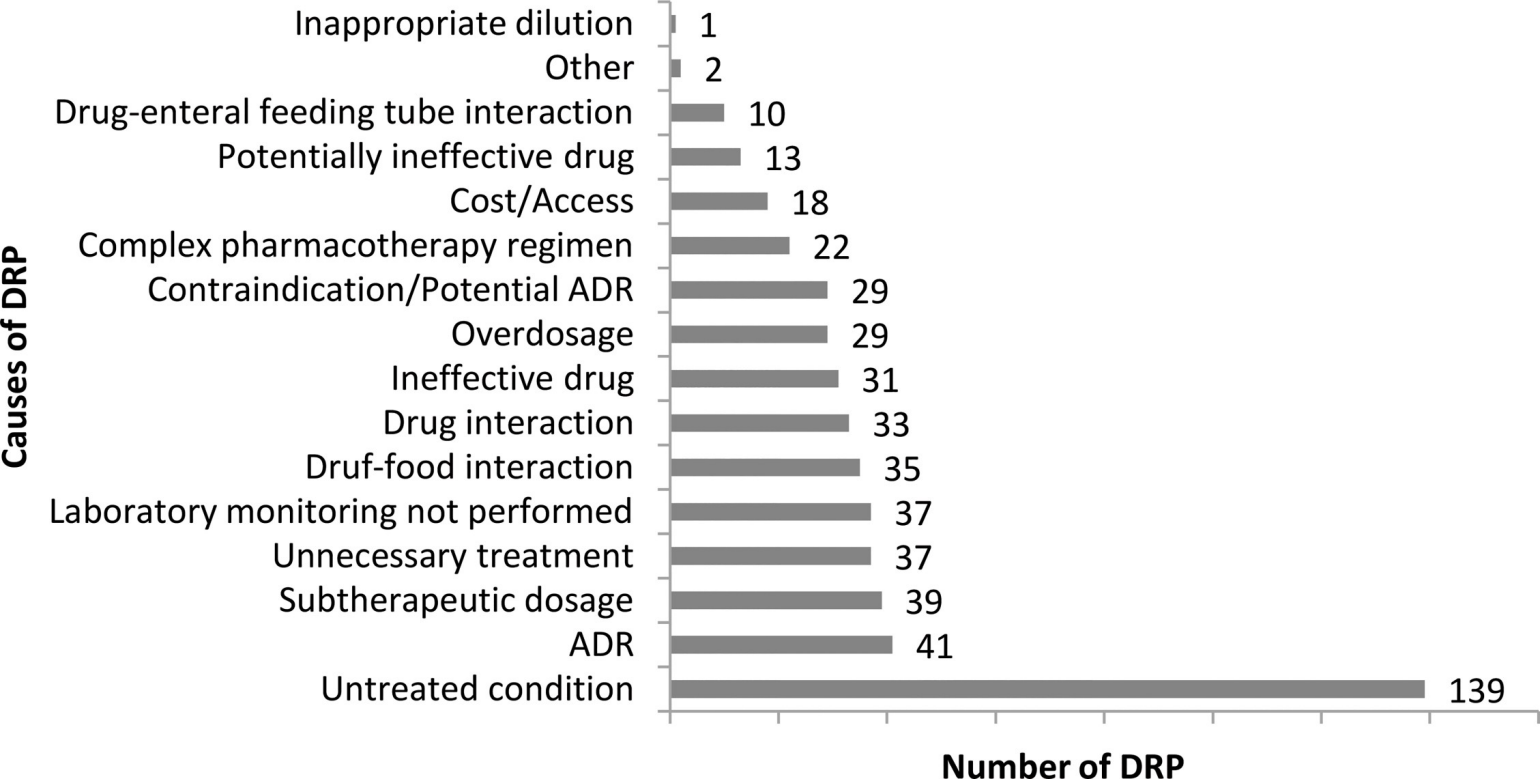
Causes of detected DRPs at the Neurology Unit of HCFMRP-USP during the study period. DRPs: drug-related problems. ADRs: adverse drug reactions.

**Fig 3:**
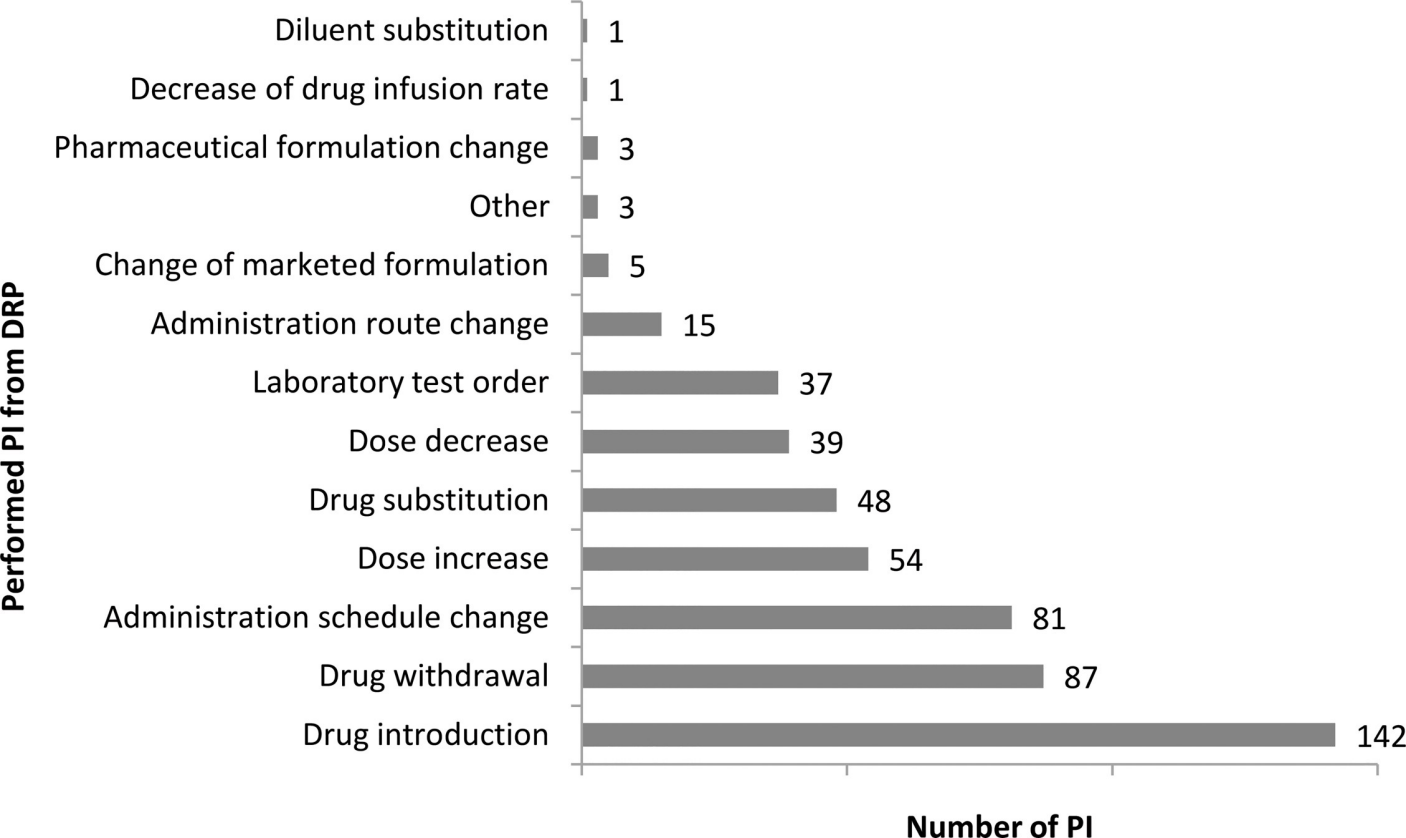
PIs that resulted from DRPs performed at the Neurology Unit of HCFMRP-USP during the study period. DRPs: drug-related problems. PIs: pharmacotherapy interventions.

Most of identified DRP was classified as "need for indication" and described as untreated condition (26.9%). Fig 2 depicts all causes of detected DRPs, as well as the number of times each was observed by the clinical pharmacist. The 516 DRPs resulted in the same number of PIs which were suggested to physicians, from which 427 (82.8%) were accepted. Regarding the moment in which the intervention that resulted from a problem was performed, 460 (89.2%) occurred during hospitalization follow-up, while 25 (4.8%) were performed by the hospital admission's medication reconciliation, and 31 (6.0%) during the discharge medication reconciliation. “Drug introduction”, most frequent PI from DRP trigger, accounted for 27.5% of these interventions, followed by "drug withdrawal" (16.9%), and "administration schedule change" (15.7%) (Fig 3). In addition to the 516 PIs described previously, there were 148 hospital discharge orientation for patients and/or caregivers. Thus, in total, the clinical pharmacist performed 664 interventions.

Drugs associated with DRP that resulted in PIs were categorized into 43 different pharmacotherapeutic classifications, the main are described in Table 2. It is noteworthy that vitamin B12 was the most frequent drug regarding PI "drug introduction" (68.4%). Considering "drug withdrawal" category, antithrombotic agents were the most prevalent (13.6%), followed by psychoanaleptics (12.5%) and drugs for functional gastrointestinal dysfunctions (12.5%). Among antithrombotics, the main observed drug was enoxaparin (66.6%), while amitriptyline and fluoxetine composed the majority of psychoanaleptics (54.5%) and bromopride was the most frequent considering the management of gastrointestinal dysfunctions (90.9%). Levothyroxine was the main drug related to the need of change on the administration schedule (13.6%).

**Table 2:** Main pharmacotherapeutic classes related to performed PIs resulting from DRPs.

ATC classification	Total PI (%*)
Vitamins	47 (9.1)
Antithrombotic agents	39 (7.6)
Antiepileptics	30 (5.8)
Psychoanaleptics	29 (5.6)
Drugs for gastrointestinal dysfunctions	27 (5.2)
Antiinfectives for systemic use	27 (5.2)
Mineral supplements	23 (4.5)
Drugs for acid related disorders	22 (4.3)
Drugs used in diabetes	22 (4.3)
Agents acting on the renin-angiotensin system	21 (4.1)
Lipid modifying agents	19 (3.7)
Drugs for constipation	16 (3.1)
Thyroid therapy	15 (2.9)
Antiinflammatory and antirheumatic products	15 (2.9)
Anti-parkinson drugs	15 (2.9)
Psycholeptics	15 (2.9)
Antianemic preparations	10 (1.9)
Calcium channel blockers	10 (1.9)

ATC: anatomical therapeutic chemical; DRPs: drug-related problems; PIs: pharmacotherapy interventions.

*Total sample size = 516.

Patients who presented any DRP had a higher mean age (mean: 53.0; SD: 16.2) in comparison with those who did not (mean: 44.5; SD: 15.7) [mean difference = -8.52; *p* < 0.001 (95% CI: -11.63, -5.40)]. However, there was no evidence of association between patient gender and the occurrence of DRP (χ2 = 3.73; *p* = 0.600). The mean period of hospitalization was 15.8 days (SD 15.0), ranging from one to 144. Those who presented DRP remained hospitalized, on average, 10.1 days longer (95% CI: 7.37, 12.88; *p* < 0.001) than individuals who have not present DRP.

According with ATC [27], most prevalent diagnoses were related to circulatory diseases [n = 198 (30.0%)], followed by endocrine and metabolic diseases [n = 176 (26.7%)], mental and behavioral disorders [n = 65 (9.9%)] and diseases of the genitourinary tract [n = 55 (8.3%)]. Regarding the circulatory system, primary essential hypertension was observed in 133 patients. Endocrine system's most prevalent diseases were diabetes mellitus (n = 58), thyroid gland disorder (n = 43), and disorders of lipoprotein metabolism and other lipidemias (n = 39). Mood and affective disorders (n = 49) stood out between mental and behavioral category, as well as urinary tract infections (n = 28) considering genitourinary tract diseases. Patients with DRP exhibited a higher mean of diagnoses per patient, 2.0 (SD 1.9), when compared to the group without DRP, 1.1 (SD 1.2), [mean difference: 0.9 (95% CI: 0.68, 1.17), *p* < 0.001].

About the etiological diagnoses related to neurological conditions that led to hospitalization, the most common diseases are described in Table 3.

**Table 3:** Most common etiological diagnoses of the neurological disorders that led to hospitalization at neurology ward.

Admitting diagnoses	n (%)
Epilepsy	44 (10.8)
Vitamin B12 deficiency	33 (8.1)
Central nervous system infections	32 (7.8)
Parkinson disease	18 (4.4)
Multiple sclerosis	16 (3.9)
Myasthenia gravis	16 (3.9)
Stroke	13 (3.2)
Drug-induced Parkinsonism/Other ADR	11 (2.7)
Motor neuron disease	10 (2.4)

The number of patients diagnosed and the frequencies of the diseases that led to at least 10 hospitalazations. ADR: adverse drug reactions.

Patients received, on average, 11.9 (SD 5.8) drugs, ranging from two to 38 drugs per patient. Those who presented any DRP showed a higher mean for this variable [14.0 (DP 6.1), ranging from 3 to 38 drugs per patient] when compared to the patients without DRP [9.3 (SD 4.3), ranging from 2 to 23 drugs per patient] (χ^2^ 181.75, *p* < 0.001).

Multiple logistic regression model (R^2^ 0.45; Hosmer and Lemeshow adherence test: χ^2^ 7.282, *p* = 0.507) indicated that each additional day of hospitalization increased the odds of presenting a DRP in 8.0% (95% CI 1.058; 1.12). The addition of a new drug increased DRP odds by 15.0% (95% CI 1.094; 1.22), while each year of life, by 2.0% (95% CI 1.003; 1.033). Moreover, inpatient with multiple sclerosis, epilepsy or myasthenia gravis have nine, two and three times more chance of presenting DRP than other patients, respectively (Table 4).

**Table 4:** Multiple logistic regression model for DRP occurrence related to: most common etiological diagnoses of the neurological disorders, length of stay, number of diagnoses, total drugs in use, and age of patients followed at the Neurology Unit of HCFMRP-USP.

Independent variable	β-coefficient	Standard error	Adjusted OR (95% CI)	*p* value
Stroke	-0.596	0.910	0.551 (0.093; 3.283)	0.513
Vitamin B12 deficiency	0.044	0.502	1.045 (0.39; 2.798)	0.930
Motor neuron disease	-0.112	0.806	0.894 (0.184; 4.338)	0.889
Parkinson disease	-0.354	0.622	0.702 (0.207; 2.377)	0.570
Multiple sclerosis	2.194	0.860	8.967 (1.661; 48.409)	0.011
Epilepsy	0.773	0.389	2.167 (1.012; 4.642)	0.047
Central nervous system infections	-0.025	0.483	0.975 (0.378; 2.515)	0.958
Myasthenia gravis	1.192	0.590	3.294 (1.037; 10.459)	0.043
Drug-induced Parkinsonism/ Other ADR	1.336	0.691	3.805 (0.982; 14.74)	0.053
Age (years)	0.018	0.007	1.018 (1.003; 1.033)	0.016
Number of diagnoses	0.123	0.090	1.13 (0.947; 1.349)	0.175
Total number of drugs used	0.144	0.028	1.155 (1.094; 1.22)	0.001
Length of hospital stay (days)	0.085	0.015	1.089 (1.058; 1.12)	0.001
Intercept	-7.336	2.181		0.001

ADR: adverse drug reaction; DRP: drug-related problem; OR: odds ratio; CI: confidence interval.

## Discussion

DRP related to indication need were the most common, particularly those classified as "untreated condition" (26.9%). Untreated health problems potentially aggravate patient's clinical condition, which may extend the length of hospitalization, consequently increasing the costs to the health system [8]. Vitamins comprised the class of drugs involved in most PIs (9.1%), from which vitamin B12 was the drug most commonly associated with "drug introduction" PI (68.4%). Vitamin B12 deficiency triggers neuronal damage and neurological disorders, such as dementia and neuropathic pain [28,29]. Yi et al. carried out a study in a neurology unit of a tertiary teaching hospital—similar reality of the present study—that corroborated this finding. The authors found that the most frequent DRPs were related to vitamin B12 deficiency that causes hyperhomocysteinemia [30]. Its early detection and treatment can prevent irreversible damage to the central nervous system, and the role of clinical pharmacist in promoting the adequate use of this vitamin is critical to the pharmacotherapy success. Interventions related to the introduction of pharmacological therapy were supported by laboratory tests, such as vitamin B12 and fasting plasma glucose, by clinical parameters, such as blood pressure values and/or by signs/symptoms indicating some untreated clinical condition. Seizures in patients with epilepsy are an example of clinical sign that may result in a “drug introduction” PI.

Antithrombotics were the second class of PI-related drugs (7.6%). In Neurology units, the hospitalization of patients with neurological conditions that are associated with motor alterations and reduced mobility is common. The indication of antithrombotics for preventing venous thromboembolism in bedridden patients who have other risk factors, such as elderly age, smoking and obesity should always be considered. Nevertheless, this class prescription should be made rationally, due to the risk of adverse reactions such as thrombocytopenia and hemorrhage [31]. This may explain the result that indicates antithrombotics as the main class involved in PI "drug withdrawal". In several cases, the pharmacist suggested withdrawing the referred drugs by judging their use was unnecessary (need for indication DRP), or due to the detection of an adverse reactions (safety DRP). PIs related to the use of antithrombotics were supported by guideline recommendations [32].

Bromopride whose use should be avoided in subjects with Parkinson's disease due to its antidopaminergic effect on the central nervous system [33], and amitriptyline, which is inappropriate for the elderly especially because of its anticholinergic effects [34], were other drugs frequently related to PI "drug withdrawal", as a consequence of a DRP related to safety. Recently published articles discuss the unnecessary use of drug therapies in the elderly [35–37]. Polypharmacy and inappropriate use of drugs are common among older people and can associate with geriatric syndromes, cognitive deficit and increased mortality. Therefore, deprescribing is the planned and safe withdrawal of drug that is not indicated to the current treatment of patients who are in inappropriate use of drugs [37].

“Drug substitution” was another common PI that frequently involves venous thromboembolism prophylaxis therapy. Enoxaparin is generally indicated in HCFMRP-USP, due to its simpler and safer dosage schedule in relation to unfractionated heparin, other available option in the hospital. However, if a significant alteration in renal function is observed, fractional heparin (enoxaparin) should be shifted to unfractionated heparin [38]. Through a prospective study—realized in a tertiary teaching hospital in London—that analyzed clinical pharmacists' interventions on venous thromboembolism prophylaxis, Lee et al. demonstrated a significant positive clinical and economic impacts [39].

Levothyroxine accounted for 13.6% of all "administration schedule change" PI, which, in turn, was the third most prevalent PI (15.7%). Administrating levothyroxine with food reduces drug absorption and may lead to drug ineffectiveness and hypothyroidism decompensation [40–42]. It is important to note that this study was carried out in a highly specialized unit, coordinated by neurologists and neurosurgeons, which may partly justify the high number of PIs related to drugs that treat diseases of another nature.

Despite this study setting, most prevalent diseases referred to disorders of the circulatory and endocrine systems. These results reflect epidemiological data regarding general adult population in most countries which show a high prevalence of diseases, such as hypertension and diabetes mellitus [43,44].

Multiple logistic regression showed that age, number of drugs used, length of hospital stay, diagnosis of epilepsy, multiple sclerosis and myasthenia gravis were factors associated to DRP (Table 4). Patient's age is an important variable and should be considered when planning the pharmacotherapy, once the aging process results in both pharmacokinetic and pharmacodynamic changes on drugs' metabolism, which interferes with the drugs effectiveness. It is also important to highlight that polytherapy increases the risk of DRP [45,46]. This may be related to the increased risk of occurring an adverse reactions for each new drug added in the treatment, as well as the greater potential of drug interactions. Considering hospitalization period, this relates to DRP in two different ways, depending on the perspective of cause-consequence binomium. That is, the occurrence of DRP may prolong hospitalization due to its management; and a longer stay, in turn, often require the indication of new drugs, that may result in a new DRP, as elucidated in our results. Although the number of diagnoses was different between the groups (with and without DRP), this may consist in a confounding variable (p = 0.261), probably due to the fact that patients with the highest number of diagnoses were older and using more drugs because of the greater number of diagnoses.

Polypharmacy is a worldwide reality when it comes to drug use reports. The mean number of drugs used by the patients (11.9; SD 5.8) was higher than those found in other two studies undertaken in hospitals whose average numbers of drugs per patient were 6.3 and 7.9, respectively [47,48]. The therapy of epilepsy involves drugs such as carbamazepine, phenytoin, phenobarbital, valproic acid and benzodiazepines that have a known potential for drug interactions and adverse events that limit the quality of life in patients with this neurological disease [13]. Therefore, PIs related to antiepileptic drugs use are common. Myasthenia gravis and multiple sclerosis are autoimmune diseases whose pharmacological treatment is generally performed with corticosteroids, immunosuppressants, immunomodulators or monoclonal antibody drugs. The use of these classes of drugs is associated with important adverse events, including those that result from their effects on the immune system [49,50].

The percentage of acceptance of PIs performed to the physicians was 82.8%, while published data vary between 70.0% and 100.0% [51–54]. Based on this, it was considered that these PIs were assertive and feasible for clinical practice in the Neurology Unit. Although meetings and multidisciplinary discussions are part of the routine neurology unit—study setting—all PIs were taken to the physician. The physician is the only professional who has autonomy to change the prescription in HCFMRP- USP.

Most of PIs were performed during the follow-up of inpatients (89.2%). Spalla and Castilho have described that either the omission or the absence of prescribing medications which are being used by the patient at home, prior to hospitalization, may represent from 42.0 to 60.0% of all errors occured during hospital admission and discharge [47]. These results suggest that medication reconciliation represents a conduct that can minimize these problems, since the pharmacist's responsibility at these two specific moments is, fundamentally, the detection of errors such as omission of drugs.

It is noteworthy that the clinical pharmacist performed his clinical activities for six hours a day during this study, from Mondays to Fridays. The aforementioned CPS routine contributed to the fact that most PIs were performed during the hospital follow-up, since the absence of the pharmacist did not allow his effective participation in a considerable number of both medication reconciliations and discharge advices. This situation contributes to possible underestimated number about reconciliation-related DRPs in comparison to other studies. Another limitation of this study was the impossibility to evaluate the impact of both PIs and CPS on patients health and quality of life after hospital discharge. In addition, the influence of admitting diagnosis severity on the occurrence of DRP was not assessed in this study. Finally, it was not possible to perform a randomized controlled study which would allow comparing the outcomes with a control group (without CPS) due to the hospital routine regarding the activities of the clinical pharmacist. Despite the cited limitations, the design of this study represents the real-life context and shows evidence the relevance and acceptability of CPS in the hospital routine.

## Conclusion

DRPs were quite prevalent in patients admitted to the Neurology Unit of HCFMRP-USP, especially among elderly patients and in the presence of politherapy. However, monitoring the patients clinical evolution and the use of drugs allowed the clinical pharmacist to detect DRPs and to suggest interventions that contributed to the optimization of pharmacotherapy and was well accepted by physicians.

## Supporting information

S1 Checklist Trendstatement_TREND_Checklist.dot. (DOT)
